# Observations on Rectal Surgery1Read before the Niagara University Alumni, Buffalo, N. Y., May 16, 1895. By invitation.

**Published:** 1895-09

**Authors:** J. M. Mathews

**Affiliations:** Louisville, Ky., Professor of surgery and diseases of the rectum, Kentucky School of Medicine


### BUFFALO MEDICAL JOURNAL.

VOL. XXXV. SEPTEMBER, 1895. No. 2.

### Original Communications.

### OBSERVATIONS ON RECTAL SURGERY.[1]

BY J. M. MATHEWS, M. D., Louisville, Ky.,

Professor of surgery and diseases of the rectum, Kentucky School of Medicine.

WHEN the invitation came to me to read a paper before this association I must confess that there seemed to be no time at my disposal. But having been in attendance at the American Medical Association, in Baltimore, I extended my trip to the east, in order to be with you tonight. I have thought in selecting a subject for the occasion that it was the proper thing for the shoemaker to stick to his last, so I propose to take some of your valuable time in discussing :

*Some Points in Rectal Surgery.*—Eighteen years ago I first conceived the idea, and after much hard work put it into execution, of making diseases of the rectum a specialty. At that time there was no such specialty either in this country or in Europe. Since then some one or more distinguished surgeons, in every large city of the union, have adopted the specialty. This, of course, is gratifying to me. I shall reserve the right in this paper to express an individual opinion upon all subjects touched upon, and in differing from others I mean no disrespect and do not wish to appear either dogmatic or egotistical. I shall refer in the paper only to those points that are mooted.

*Hemorrhoids.*—Authors who have written specially on diseases of the rectum have divided hemorrhoids into two main classes : first, external ; second, internal ; and then have subdivided the classification into varieties of two external and three of internal. Of the first, a tag of skin or a thrombotic tumor go to make up this class. Of the second variety, such terms as arterial, venous

1. Read before the Niagara University Alumni, Buffalo, N. Y., May 16, 1895. By invitation.

and capillary are used to express them. Now, in truth, it makes very little difference with the operator whether piles are classed at all. For convenience sake it would be much better to say, with Mr. Erichsen, "all external piles should be cut off and all internal piles tied." In my opinion the universal advice given, to let out the clot of a thrombotic pile, is non-surgical. After such an incision is made it takes the inflamed tissue which is left just as long to disappear by reabsorption as it would have taken nature to absorb the clot. In a word, then, all external piles should be cut off. A clean even base is left, and the patient is well in one-half the time when compared to the other plan.

*Internal Piles.*—It matters very little with the operator whether internal piles are venous, arterial or capillary in nature. To diagnosticate such would not alter the operation in the least. Again, the classification is erroneous, for the reason that no hemorrhoid is made up exclusively of arteries, veins or capillaries. As to the method of ligating piles, I object to the plan usually named as that of Mr. Allingham, but is the one proposed by Mr. Salmon—namely, that the cut should be in the sulcus, or white line, and carried down to the base of the pile, and after ligating, that but a small portion of the pile should be cut off. If superfluous skin exists around the anus, it is much better to include it all in the incision made and by the ligature used. If such mass is transfixed and tied tightly, it should be cut off in such a manner as to leave but a small base to slough. The idea that a contraction takes place after such an operation is chimerical, and even if it did, one dilatation with the speculum would overcome it. After operating upon over 2,000 patients for piles, I have never encountered contraction but in two cases, and they were relieved in the manner described. I have said that all internal piles should be tied, because my experience has taught me that compared with all other methods the ligature is the simplest of execution, freer of danger and most radical in its results.

*Fistula in Ano.*—This term does not in fact bear out the significance intended. Very few fistulæ in this region relate to the anus at all. Many of them communicate with the rectum, and some fistulæ in this neighborhood neither affect the anus or rectum. A buttock may be extensively invaded, the perineum destroyed, or a sinus run high up the back, when it will not be necessary to follow the time-honored injunction to push a director through the sinus into the rectum and divide all the tissues thereon, including the sphincter muscle or muscles. Indeed, such a procedure would be out of the question in many of these cases. From time immemorial anal fistulæ have been divided into three varieties—namely, external, internal and complete. Great stress is laid upon the importance of finding the internal opening of a fistula before operating. So far has this fallacy gone that I have known surgeons to refuse to operate for fistula in ano because the internal opening could not be found. This is sheer nonsense. After the main cut is made for fistula, how easy it is to trace the channel to an internal opening, if one exists. I desire to say in this connection that no idea whatever can be conveyed to the surgeon by the appearance of the external opening of a fistula. Some of the most serious cases that I have ever operated upon were those having a small, insignificant opening. Many times a whole buttock will have to be sacrificed to effect a cure in such cases. The etiology has much to do with determining the method of operating, looking to its cure or eradication.

To classify the disease simply by physical signs, such as external, internal and complete, gives but a faint idea of its nature. Or to describe a fistula as a narrow channel, or sinus, lined with a so-called pyogenic membrane, is so far fetched as not to deserve recognition. No membrane ever secreted pus, and yet this term *pyogenic* is still used by some writers. Again, very few fistulæ are ever seen which are made up of a narrow channel or sinus. If such terms should be taken as guides in estimating the amount of disease, or mapping out an operation, not one in fifty cases would ever be cured. A far better division of fistula in ano, in my opinion, would be,—namely, progressive and non-progressive. Some fistulæ can be left without an operation for an indefinite time. Others are so distinctive and destructive in their nature as to require immediate attention. Instead of mere sinuses in the region of the rectum, well-formed cavities with a continued disposition to break down tissue will often be found. Outside of the risk of sepsis in such cases, the local destruction may be so great as to render the individual a cripple or an invalid for life. It is a well-recognised fact that abscesses around the rectum, in a heavy percentage of cases, end in fistulæ. Many account for this fact by the statement that the circulation here is impeded by the absence of valves in this portion of the blood circulation. But a factor of far greater importance is overlooked—namely, that the tissues being soft, fatty and often flabby, are unable to resist the inflammatory process. Besides, it must be remembered that certain diatheses, or cachexias, render these tissues peculiarly susceptible to the destructive process of inflammation. But even in healthy tissue the inroad from the inflammatory deposit is often such as to make an active and destructive condition that, unless heeded at once, ends in a serious complication of affairs. With this idea in mind, I would suggest some things which I think necessary in the successful treatment of this very troublesome affection. Speaking from a large and varied experience in surgery, I desire to say that it requires more delicate and precise operating to cure a complicated condition of fistula in ano, than any other surgical affection of which I can think. In referring to the treatment of fistula I shall have nothing to say of the use of caustics, injections, ligatures and the like, proposed by some for the cure of this affection. They are, to say the least of them, unsurgical and unsatisfactory. It is well known that there are comparatively few cases of fistula in ano that are limited to a single tract. Indeed, the most prominent thing that should be brought out in operating for this trouble, is the laying open of all communicating branches. This can only be done, of course, by the use of the knife. It is too common with the profession to think of fistula in ano as simply an opening from the true skin down to or into the bowel. The fact is that all adjacent parts to the rectum may be undermined or invaded by these fistulous channels. It can be easily understood, looking to the pathology of this affection, that no treatment can compare with the knife in dealing with it. By its use all sinuses can be divided, the bottom of fistulæ cut through and overhanging edges trimmed away, these precautions being absolutely necessary to a cure in such cases. The old idea that fistula in ano acted as a derivative in tubercular affections of the lungs is, of course, obsolete and appears in a ridiculous light today. Therefore, the early eradication of the trouble, even in tubercular subjects, is much to be desired, and which can often be accomplished by an operation.

*Ulceration of the Bowel.*—I am constrained to believe, from my correspondence with physicians and the knowledge gained in consultation, that the opinion prevails that ulceration of the rectum (benign) is of the most frequent occurrence. I wish to go on record as saying that it is the most infrequent of all rectal troubles. Indeed, in eighteen years of experience in this line, I have not seen half a dozen cases of ulceration of the rectum due to simple causes. This alone would force me to believe that the statement of writers that this trouble is a frequent occurrence is chimerical. Mucous membrane is very seldom the seat of ulceration, except it be of a special diathesis. So well pronounced are my views on this subject that I desire to state without argument, whenever I see a well-pronounced case of ulceration of the rectum I am at once convinced that it has its origin in one of three conditions—namely, syphilis, tuberculosis or malignancy. As I have put myself on record in my work on Diseases of the Rectum, as saying that such causes of ulceration of the rectum as dysentery, pressure of the child's head, constipation, and the like, are of the rarest occurrence, I would respectfully ask that the members of this society call up any such cases in their minds and report them. So well persuaded am I that all such combined are insignificant as a cause of ulceration, that whenever I see an ulcer of a pronounced type in this portion of the gut, I know that it is of a much more serious origin than from any such causes as these. The three great factors, therefore, in my opinion, in producing ulceration of the rectum are tuberculosis, syphilis and cancer. With either one as a cause, ulceration is a grave affair. If stricture of the rectum should result from any cause outside of these, it is easily remedied. It must be seen that such a stricture could only involve the mucous membrane, and would be annular in appearance. A simple dilatation would effect a cure. But a very different condition of affairs would result from either one or the other of the three mentioned causes. In other words, a stricture from pressure, trauma, dysentery, and the like, would be a benign or simple stricture. Whereas from the last mentioned—namely, tuberculosis, syphilis or cancer—the stricture would be secondary to a constitutional affection. We are too apt to look for a demonstration of tuberculosis in the lungs, possibly in a joint, when, from a later study of the disease, it is recognised that the tubercular bacilli may have as a starting point any tissue, or tissues, of the body. Notably is this true of the rectum. My record books show many cases where tuberculosis has attacked the rectum and left other tissues unaffected. In regard to syphilis as a cause of ulceration, with consequent stricture, I have before this given it as my opinion that fully *sixty per cent.* of such cases result from this cause. I only ask a careful compilation of your cases to verify this statement. In this connection I wish to give it as my opinion that stricture of the rectum resulting from syphilis is just as incurable as cancer, the only difference being that cancer kills more speedily, and that the sufferer from syphilitic stricture has a longer lease on life. Strictures from cancer often exist without the manifestation of the pronounced symptoms that we usually expect. Indeed, there are many cases recorded from my practice where only constipation was complained of, with some reflex pain in contiguous organs. Of course, this only holds good when the growth is located above the sphincter muscle and does not involve it.

*Treatment.*—Of course, I can only suggest the manner or method of treatment of ulceration of the rectum, not having the time to enter into a full description of any plan. For a tuberculous ulceration there can be but one method to suggest. In the light of modern research it must be conceded that tubercular disease can have its origin or starting point in any tissue. The lungs may be secondary, in this trouble, to any other portion of the body. The surgeon meets with a tuberculous knee-joint, he excises it; if with tuberculous bone, he removes it, not only for its local effect, but from the knowledge that a general infection can take place from such foci. Therefore, if a tuberculous ulceration of the rectum exists, it must be thoroughly curetted. I am confident that I have prevented a general infection in a number of cases by a full and complete curettement of the rectum. Of the syphilitic rectum nothing so good can be said. Whatever may have been the opinion of pathologists a decade ago, it must be agreed today, if syphilis affects the rectum, it is by a secondary deposit,—by a gummatous building-up process. This assumes a fibrous condition beyond the power of nature to reabsorb. We must, therefore, accept the ultimatum and do the best we can. I believe that the landmarks are plain enough and the physical signs sufficient to draw the diagnostic line between strictures from this cause and the other sources I have mentioned. I might concede that, if syphilitic ulceration of the rectum was detected in its incipiency, constitutional medication might effect some good. But not so with the condition of which we are speaking.

When a decided stricture exists it becomes a matter for surgical interference. What shall it be? I am familiar with the fact that some writers deal with all serious ulcerations or strictures of the rectum in one of two ways surgically, *i. e.*, total excision or colotomy. I am averse to both. If syphilis has affected the lower rectum, and results in stricture, it certainly appears advisable to divide or break the stricture and avoid the opening in the side. Especially is this true when we recognise that the process of infiltration is more or less self-limited, and we have nothing to fear in the extension of the disease except the obstruction. A free proctotomy in such cases is much better than a colotomy. If a stricture, the result of syphilis, exists in the movable or the upper rectum, I believe that it is an ideal case for a colotomy. It is a localised condition without the power of extension; nature cannot absorb it, and obstruction results, which to all intents and purposes is purely mechanical. If it is dangerous to dilate, an opening can be made above, and the patient live indefinitely. How different from a cancerous stricture! The destructive process goes on, ends in death, regardless of the colotomy. In cancer of the rectum, or cancerous stricture, the two methods mentioned—namely, total extirpation or a colotomy—is resorted to. First, let us consider the propriety of total excision. With so many to recommend it, I feel some diffidence in entering my protest against it. Mr. Kraske has designed a most excellent operation for the removal of the rectum. But I would beg to ask, When is the operation justifiable? There are two propositions that I would submit for argument in reasoning against this operation. First, is the patient ever cured of the trouble operated for by said operation? Second, is the patient ever materially benefited or relieved by the operation? I beg to take the negative to both propositions. First, I take it for granted that the only cause for which it could be said that total extirpation could be advised is malignancy. I use the term synonymous with cancer. We note, then, the characteristic features of malignancy as follows: the disposition to grow, to ulcerate, to infiltrate and to propagate. It is an aphorism well known in surgery in cases of cancer, to "operate early if you desire success." All teachers, too, know how assiduously they teach the student to do a thorough operation, and "cut wide of the mark" whenever dealing with this character of tumor. For instance, when removing malignant growths of the breast, how important it is, we say, to remove the glands in the axilla also. Again, how insidiously does a cancer invade the system and establish a cachexia. How difficult to tell the dividing line between a local condition and a general infection! Add to this the suspicion always that the tumor has likely propagated. These questions are difficult of solution, even when the growth is situated upon the external parts. How much more difficult when located in an obscure part of the anatomy, as in the rectum! Cancers found here are so stealthy in their growth and in the invasion of tissue that oftentimes the whole rectum is blocked by the mass, when only symptoms of constipation and some reflex pain are complained of. Tissues surrounding this portion of the gut are soft and easy of invasion, the blood circulation is great, the lymphatics are freely distributed. The contiguous parts are vital ones—the bladder, peritoneum, the prostate gland and the vagina in the female, are all easy of invasion. By the time that a growth in the rectum has assumed sufficient proportions to be diagnosticated as cancer, many of these parts are already invaded by the process of infiltration. To dissect and draw down the rectum, in the normal state, may be comparatively an easy matter, but to completely dissect out a rectum that is so pathologically changed, as in a cancerous growth, is nearly an impossibility. Besides the difficulty of accomplishing its free dissection, it is one of the bloodiest operations that I have ever attempted. Admit that it can be successfully done, what amount of good accrues to the patient? Infiltration is clearly proved by the amount of adhesion that is witnessed. Can we now "cut wide of the mark?" I believe that every surgeon present will concur in the opinion that to make the operation complete all glands in the inguinal region should be removed. But what about the infiltrated tissue left in and around the rectum? Then, of what use is the operation? A bloody procedure, rectum gone and infiltrated tissue left. Would any sensible surgeon dissect out a cancer in the breast and cover the wound with infiltrated flaps? Why, then, should a surgeon dissect out a cancer of the rectum and leave infiltrated tissue? If a growth of the kind is situated in the lower part of the rectum, of course it can and should be excised, for we take the precaution here to "cut wide of the mark." It may be said that after excision the patient lives for several years (?).

I shall not take your time to enter into an argument *pro* or *con* concerning colotomy for the relief of cancer of the rectum, but will make it suffice to offer a few suggestions for your consideration. It must be a well-recognised fact that in the majority of cancers of the rectum located above the sphincter muscle, pain is not a factor. It must also be admitted that, as a rule, these patients do not suffer from obstipation to any marked degree. Why, then, do a colotomy for the relief of pain that does not exist, or for an obstruction that oftentimes does not take place?

No one will contend that by doing a colotomy, cancer of the rectum is either cured or its progress materially stayed. If an obstruction does exist and is within reach of the finger, it is much better to do a proctotomy than to open the side. It is a disgusting operation at best, promising very little, and should be avoided if possible.

